# Proteomic profiling of extracellular vesicles reveals additional diagnostic biomarkers for myocardial infarction compared to plasma alone

**DOI:** 10.1038/s41598-019-45473-9

**Published:** 2019-06-20

**Authors:** Olof Gidlöf, Mikael Evander, Melinda Rezeli, György Marko-Varga, Thomas Laurell, David Erlinge

**Affiliations:** 1 Department of Cardiology, Clinical Sciences, Lund University, PO Box 118, 221 00 Lund, Sweden; 2 Department of Biomedical Engineering, Lund University, PO Box 118, 221 00 Lund, Sweden

**Keywords:** Proteomics, Mechanisms of disease, Diagnostic markers, Myocardial infarction

## Abstract

Extracellular vesicles (EVs) are submicron, membrane-enclosed particles that are released from cells in various pathophysiological states. The molecular cargo of these vesicles is considered to reflect the composition of the cell of origin, and the EV proteome is therefore a potential source of biomarkers for various diseases. Our aim was to determine whether EVs isolated from plasma provide additional diagnostic value or improved pathophysiological understanding compared to plasma alone in the context of myocardial infarction (MI). A panel of proximity extension assays (n = 92) was employed to analyze EV lysates and plasma from patients with MI (n = 60) and healthy controls (n = 22). After adjustment for multiple comparisons, a total of 11 dysregulated proteins were identified in EVs of MI patients compared to the controls (q < 0.01). Three of these proteins: chymotrypsin C (CTRC), proto-oncogene tyrosine-protein kinase SRC (SRC) and C-C motif chemokine ligand 17 (CCL17) were unaltered in the corresponding plasma samples. As biomarkers for MI, rudimentary to no evidence exists for these proteins. In a separate group of patients with varying degrees of coronary artery disease, the decrease in EV-associated (but not plasma-related) SRC levels was confirmed by ELISA. Confirmation of the presence of SRC on EVs of different sizes and cellular origins was performed with ELISA, flow cytometry and nanoparticle tracking analysis. In conclusion, the data revealed that despite a similarity in the EV and plasma proteomes, analysis of isolated EVs does indeed provide additional diagnostic information that cannot be obtained from plasma alone.

## Introduction

Extracellular vesicles (EV) are a heterogeneous group of membrane-limited particles released from cells upon stress, activation or injury^1^. The major EV family members are exosomes and microvesicles (MV). Exosomes are 40–100 nm particles that are formed in intracellular multivesicular bodies (MVBs) and released upon exocytosis of the MVB. MV are 100–1000 nm in size and are released by budding of the plasma membrane. EVs contain a wide array of proteins, lipids and miRNAs: and the specific composition thereof is believed to reflect the state of the cell of origin. As such, EVs are considered to be rich source of biomarkers for various disease states^2,3^.

In the context of cardiovascular disease (CVD), it has been shown that plasma EVs are associated with various cardiovascular risk factors, including smoking^4^, dyslipidaemia^5,6^, diabetes^7^ and hypertension^8^. Experimental studies have shown evidence that EVs can affect the structure and composition of atherosclerotic plaques^9,10^. Moreover, different EV subpopulations increase after a myocardial infarction (MI) and levels thereof have been shown to be independent predictors of cardiovascular outcome in patients with heart failure and coronary artery disease^11^. Despite the wealth of studies associating EV levels to different pathophysiological aspects of CVD, there are relatively few reports that have investigated the contents of these vesicles in CVD patients. In particular, the EV proteome is largely understudied. Mayr *et al*. performed a comprehensive characterization, including proteomics, of microvesicles from human atherosclerotic plaques^12^. Banfi *et al*. investigated the proteome of endothelial cell-derived microvesicles^13^ and Velez *et al*. surveyed the proteome of plasma vesicles from patients with ST-elevation myocardial infarction (STEMI) patients and stable coronary artery disease^14^. In addition, we have previously compared the proteomic profiles of plasma EV from patients after an MI with those from healthy controls^15^. These studies however, all utilized mass spectrometry-based approaches that are known under such circumstances to have limited sensitivity and a bias towards abundant proteins^16^. To obtain a deeper, comprehensive representation of the EV proteome (including proteins of lower abundance), a more targeted and sensitive approach is required. The proximity extension technique allows multiplex detection of low-abundance proteins (i.e. present in plasma at concentrations < 10 ng/ml^17^) in small sample volumes^18^. In the present study, plasma EVs were isolated from MI patients and healthy controls with acoustic seed trapping^19^ and the levels of > 90 low-abundance proteins were analyzed with proximity extension assays (PEA). As a reference, platelet-depleted plasma from each patient was also analyzed using the same method. Our aim was to compare the proteomic profiles of EVs and plasma, identify biomarker candidates that were unique to EVs, evaluate the diagnostic accuracy of these biomarkers and characterize these with respect to size and cellular origin.

## Methods

### Study cohort

ST-segment elevation myocardial infarction (STEMI) patient plasma (n = 60) was obtained from the SWEDEHEART biobank. Here, peripheral venous blood samples from patients admitted to the coronary care unit for suspected MI are stored. Dependent on subsequent examination during hospitalization, patients were later adjudicated for a final diagnosis. Peripheral venous blood samples were also collected from healthy individuals (n = 22). Patient information are summarized in Table 1. For validation, plasma samples from patients diagnosed with STEMI, unstable angina pectoris (UAP) and stable angina pectoris (SAP) were obtained from the LUNDHEARTGENE biobank. Patient information for the validation cohort is summarized in Table 2. All patients gave written, informed consent. The study was approved by the Lund University Ethics Committee and followed the recommendations of the Declaration of Helsinki. Cell-free plasma was prepared by centrifugation at 1,600 × *g* for 15 minutes and stored at −80 °C.

**Table 1:** Patient information – Discovery cohort.

Characteristic	STEMI	CTRL	p
Sample Size	60	22	
Age	66.0 (13.3)	63.7 (9.3)	0.458*
Male sex (n [%])	40 (66%)	18 (82%)	0.274**
Troponin T (ng/μl)	3528.4 (4670.5)	—	—
CRP (mg/l)	12 (68.2)	—	—
Creatinine	88.7 (28.8)	—	—
Cys C - eGFR	70.3 (18.8)	—	—
Total Cholesterol (mM)	4.9 (1.1)	—	—
HDL (mM)	1.1 (0.4)		
LDL (mM)	3.1 (0.94)		

Information of participants included in the discovery cohort. Continuous variables are presented as mean and standard deviation. CRP, C-reactive protein; Cys C, cystatin C; HDL, high density lipoprotein cholesterol; LDL, low density lipoprotein cholesterol. *Determined with an unpaired t-test. **Determined with Chi-Square and Fisher’s exact test.

**Table 2:** Patient characteristics – Validation cohort.

Characteristic	STEMI	UAP	SAP	p
Sample size	8	8	8	
Age	65.5 (9.9)	64.0 (8.2)	65.9 (8.1)	0.90*
Male sex (n [%])	7 (87.5)	6 (75.0)	6 (75.0)	0.78**
Troponin T (ng/ul)	139.3 (118.2)	55.6 (100.1)	2.8 (0.6)	**0**.02*
CRP (mg/l)	21.9 (49.9)	6.9 (2.1)	19.7 (24.1)	0.61*
Creatinine	83.8 (15.7)	99.0 (44.5)	90.3 (32.2)	0.43*
Total Cholesterol (mM)	5.2 (1.3)	5.2 (1.5)	4.2 (0.8)	0.19*
HDL (mM)	1.1 (0.2)	1.2 (0.3)	1.4 (0.5)	0.25*
LDL (mM)	3.2 (0.9)	3.3 (1.3)	2.0 (0.9)	**0**.04*

Information of participants included in the validation cohort. Continuous variables are presented as mean and standard deviation. CRP, C-reactive protein; HDL, high density lipoprotein cholesterol; LDL, low density lipoprotein cholesterol. *Determined with one-way ANOVA. **Determined with Chi-Square and Fisher’s exact test.

### EV isolation by acoustic seed trapping

The acoustic trapping platform has been described previously in detail^20–22^. Briefly, samples are aspirated into a rectangular 2 × 0.2 mm^2^ borosilicate capillary to which a 4 MHz PZT transducer has been attached. The transducer is used to generate a local λ/2 acoustic standing wave in the capillary. The resulting pressure and velocity amplitude gradients are used to retain 12 μm polystyrene beads (so called seed particles) against flow. Submicron particles (e.g. EVs) in the sample will then be trapped in the vicinity of the seed particles by secondary acoustic forces. For this study, an automated configuration combining an acoustic trapping unit and a robotic 96-well plate was used (AcouTrap, AcouSort AB, Sweden). 100 μl of plasma was diluted 1:2 in phosphate-buffered saline (PBS) and aspirated at 30 μl/min into the previously-trapped seed particle cluster. While retained in the trap, the EVs were washed with 50 μl of PBS. Soluble protein in the trap after washing was measured spectrophotometrically at 280 nm using Nanodrop and was shown to be reduced >10-fold compared to input plasma (Supplementary Fig. 1). Trapped samples were released in 30 μl of PBS. Processing time for acoustic trapping was approximately 7 minutes/sample. Radioimmunoprecipitation assay (RIPA) buffer was directly added to the EV suspension and the samples were incubated for 10 minutes at 4 °C. The lysates were cleared by centrifugation at 8000 × *g* for 10 minutes at 4 °C and stored at −80 °C.

### Proximity extension assay

Using the Proseek Multiplex CVD I96 × 96 panel (Olink Proteomics, Uppsala, Sweden), proteomic profiling was performed at the Clinical Biomarkers Facility at the Science for Life Laboratory, Uppsala University, Sweden. The panels included a total of 92 proteins that had been selected based on the role played in cardiovascular disease (Supplemental Table I). The proximity extension method is a highly-sensitive, multiplex immunoassay particularly suited in profiling and quantitating low-abundance-proteins^18^. Briefly, the technique is based on the binding of two oligonucleotide-conjugated antibodies that, upon binding to their respective epitopes on the target protein, form a target sequence for a quantitative real time PCR (qRT-PCR) reaction. The qRT-PCR data are transformed into normalized protein expression (NPX) units using the following equations:

$${{\rm{Ct}}}_{{\rm{Analyte}}}-{{\rm{Ct}}}_{{\rm{Extension}}{\rm{control}}}={{\rm{dCt}}}_{{\rm{Analyte}}}$$

$${{\rm{dCt}}}_{{\rm{Analyte}}}-{{\rm{dCt}}}_{\mathrm{Inter}-\mathrm{plate}{\rm{Control}}}={{\rm{ddCt}}}_{{\rm{Analyte}}}$$

$${\rm{CF}}-{{\rm{ddCt}}}_{{\rm{Analyte}}}={{\rm{NPX}}}_{{\rm{Analyte}}}$$

The extension control is composed of an antibody coupled to DNA-tags that are always in proximity and therefore results in a constant signal independently of the immunoreaction. The Inter-plate controls are included in triplicates on each analysis plate and consists of a pool of 92 antibodies with fixed-proximity DNA-tags, which can be viewed as a synthetic sample with a high signal. The median of the IPC triplicates is used to normalize each assay, compensate for potential variation between runs and plates. The correction factor (CF) is pre-determined using a negative control sample and is used to invert the scale so that a higher value corresponds to a higher signal and that the background levels are approximately zero.

A protein was defined as detected if the NPX exceeded the limit of detection (LOD) for > 50% of samples (either in the patient or the control group). For the analysis of the data, values < LOD were replaced by LOD/2.

### Flow cytometry

Thawed plasma samples were centrifuged at 1,600 × *g* for 15 min at RT. EVs were isolated with acoustic seed trapping as described above or by centrifugation at 20,000 × *g* for 1 h and released or resuspended in PBS. SRC antibody (#2108, Cell signaling) or IgG isotype control antibody were added at a 1:200 dilution and the samples were incubated at RT for 1 h. EVs were then pelleted by centrifugation at 20,000 × *g* for 1 h and resuspended in PBS with 0.5% BSA and an Alexa Fluor 488-conjugated secondary antibody (Cell Signaling) diluted 1:1,000. The sample was incubated 30 min at RT and washed with PBS containing 0.5% BSA. The vesicle pellet was resuspended in PBS and analyzed on an Accuri C6 flow cytometer. As previously described, the EV gate was set based on a series of submicron size standard beads as described previously^19^, and the proportion of SRC^+^ events in the EV gate was assessed.

For analysis of the cellular origin of SRC^+^ vesicles, plasma samples from healthy controls were prepared as described above and in addition to SRC, were stained using PE-conjugated antibodies towards CD42a, CD62E, CD16 or an isotype control (all from Beckton Dickinson, Franklin Lakes, USA) at the concentrations recommended by the manufacturer.

### Nanoparticle tracking analysis

EVs were isolated with acoustic trapping or centrifugation and stained with a SRC antibody as described above. Samples were run on a NanoSight LM10 (Malvern Instruments, Malvern, UK) in scatter and fluorescence mode with five repeated measurements. The capture settings were as following: Camera level:13, Slider shutter: 1232, Slider Gain: 219, FPS: 25.0, number of frames: 1498 and Syringe Pump Speed: 100. Analysis settings were as follows: Detect Threshold: 5, Blur Size: Auto and Max Jump Distance: 10.9 pixels. A control sample containing only secondary antibody in PBS with 0.5% BSA was run to assess background fluorescence. Five repeated measurements was performed on each sample.

### ELISA on plasma and EVs

EVs isolated with acoustic trapping (as described above), whole plasma, microvesicle-depleted plasma (obtained by centrifugation at 20,000 *g* for 1 h) and exosome-depleted plasma (obtained by centrifugation of microvesicle-depleted plasma 100,000 *g* for 1 h) was analyzed with the Total In-cell ELISA Kit (abcam, Cambridge, UK) using the Total SRC-antibody or the Quantikine CCL17/TARC ELISA (R&D Systems Inc., Minneapolis, MN, USA) according to the manufacturer’s instructions.

### Statistical analyses

Hierarchical clustering and principal component analysis (PCA) was performed with ClustVis^23^, logistic regression was performed in SPSS v. 23 (IBM, Armonk, NY, USA) and all other statistical analyses were performed in GraphPad Prism v. 7 (GraphPad Software, La Jolla, USA).

Differential expression of proteins between MI patients and controls were analyzed using t-tests. To account for multiple comparisons, the false discovery rate (FDR) was controlled with the Benjamini and Hochberg approach^24^. FDR-adjusted p values (refered to as q-values) < 0.01 were considered significant. Correlations of NPX-values between the different sample groups were analyzed by calculating Pearsons correlation coefficients. The area under the receiver operating characteristic curve (ROC) was compared between plasma and EV isolates with the method of Hanley and McNeil^25^.

## Results

### The proteomic profiles of EVs isolated with acoustic seed trapping and centrifugation is highly correlated

We have previously shown that the proteomic composition of EVs analyzed with mass spectrometry is highly similar comparing acoustic seed trapping and high-speed centrifugation^26^ but wanted to confirm that there was no impact of EV isolation technique on the results of the proximity extension assay. To this end, we re-analyzed a previously published data set^27^ comprising a total of 30 acoustically trapped and 30 centrifuged EV samples from 10 healthy donors assayed with the Olink CVD II PEA Panel. 59 and 55 proteins were detected (i.e. signal above the limit of detection in > 50% of samples) in centrifuged and trapped EV samples, respectively. Of these proteins, 53 were common to both sample types (Supplemental Fig. 2a). We next compared the mean levels of these 53 proteins across sample types and observed a very strong correlation (r = 0.965, p < 0.0001, Supplementary Fig. 2b). Finally, we analyzed the correlation of individual samples isolated with centrifugation of trapping and observed statistically significant correlations for 94% of the proteins (Supplemental Fig. 2c). Taken together, these results demonstrate that EV isolation technique has very limited effect on the proteomic profile measured by PEA.

### Proteomic profiling of extracellular vesicles and plasma

**Figure 1:**
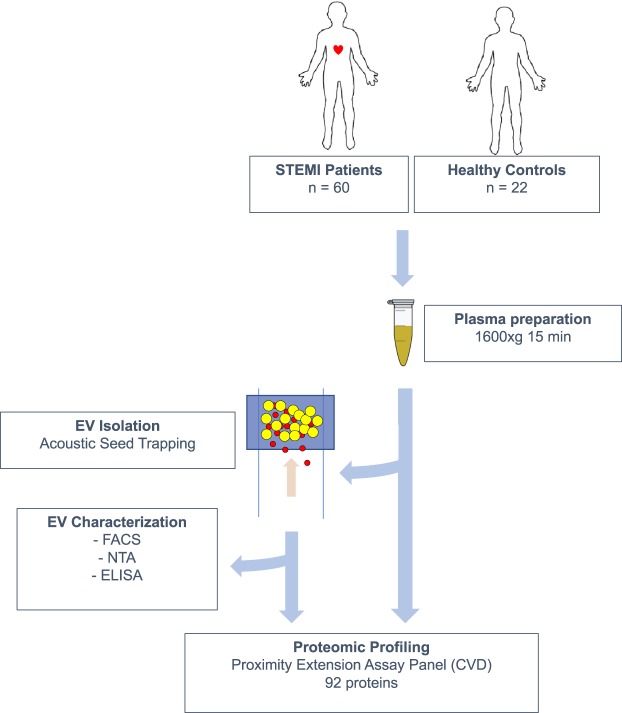
Overview of the study design. 60 STEMI Patients and 22 healthy controls were included in the study. Plasma was prepared by centrifugation of whole blood and was either analyzed directly or used for the isolation of EVs by acoustic seed trapping. Proteomic profiling was performed on whole plasma and isolated EVs using a panel of proximity extension assays with relevance to cardiovascular disease (CVD). EVs were further characterized by Flow cytometry (FACS), Nanoparticle Tracking Analysis (NTA) and Enzyme-linked immunosorbent assay (ELISA).

**Figure 2:**
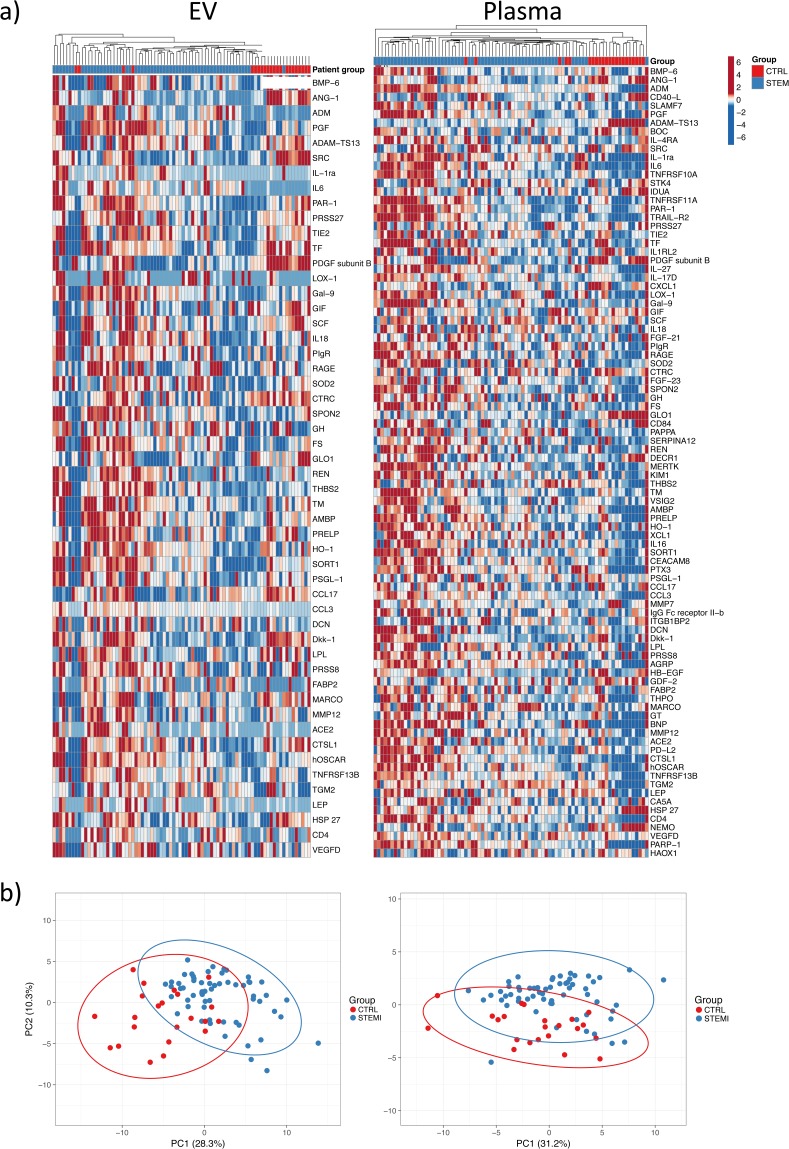
Proteomic profiling of EV lysates and plasma. (**a**) Heatmaps of all detected proteins in EV lysates (n = 52) and plasma (n = 92) across all samples. Unit variance scaling is applied to proteins and individuals are clustered using Manhattan distance and average linkage. (**b**) Principal component analysis based on all proteins detected in EV lysates and plasma. Plots show the first two principal components and their relative contribution to overall variance. Prediction ellipses are such that with a probability 0.95, a new observation from the same group will fall inside the ellipse.

An overview of the work flow for this study is presented in Fig. 1. Using the Olink CVD II PEA panel, 92 low-abundance plasma proteins that are relevant to cardiovascular disease (listed in Supplemental Table 1) were quantitated in EV lysates and plasma samples from patients with ST-segment elevation myocardial infarction (STEMI, n = 60) and healthy controls (CTRL, n = 22). Patient information is provided in Table 1. A total of 52 proteins were defined as detected in the EV lysates, *i*.*e*. present in > 50% of the samples from either the STEMI or CTRL group (Fig. 2a). All 92 proteins were detected in the plasma samples (Fig. 2a). Hierarchical clustering and principal component analysis (PCA, Fig. 2b and Supplemental Table 2) based on the levels of all detected proteins showed, with a few exceptions, that STEMI patients and CTRL individuals clustered separately. Based on these results, we conclude that a general perturbation of the EV and plasma proteome occurs during MI.

### Comparison of plasma and EV protein profiles

**Figure 3:**
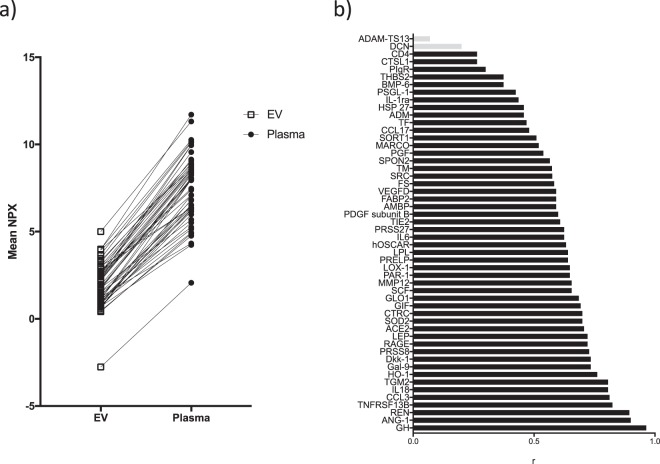
Comparison of proteomic profiles between EVs and plasma. (**a**) The mean level of the detected proteins (including both STEMI and control samples) in the EV fraction compared between sample types. (**b**) Correlation between EV and plasma samples. Shown are r-values for all proteins detected in the EV lysates. Bars in grey represent proteins with non-significant correlations (p > 0.05).

To assess whether the EV protein content differed from that in plasma, the relative levels of the 52 EV proteins were firstly analyzed and compared across the sample types. On average, the assay signal was 4-fold higher in the plasma fraction, however, the relative protein levels in each fraction was essentially identical (Fig. 3a). In addition, the majority of the EV proteins showed a robust correlation between EVs and plasma (Fig. 3b). Only von Willebrand factor-cleaving protease (ADAMTS13, r = 0.07) and decorin (DCN, r = 0.21) did not show statistically significant correlations. Based on these results, the conclusion that was drawn is that, across the low-abundance proteins detected in both fractions, the general profile of isolated EVs is similar to plasma.

### Identification of differentially-expressed proteins in patients and controls

**Figure 4:**
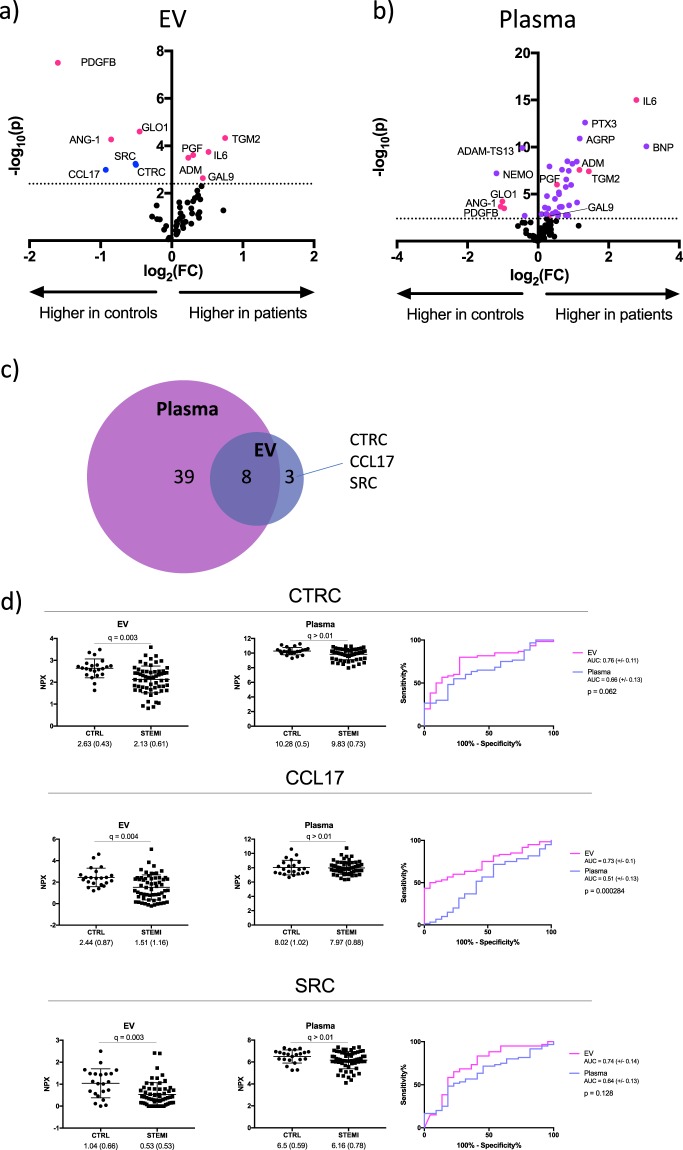
Identification of dysregulated proteins in STEMI patients. Volcano plots showing differentially-expressed proteins (q < 0.01) in (**a**) EV lysates and (**b**) plasma. Proteins differentially-expressed in both EV lysates and plasma (pink); proteins differentially-expressed only in EV lysates (blue) or only in plasma (purple). The dotted line represents the threshold for statistical significance. (**c**) Venn diagram of differentially-expressed proteins. (**d**) Scatter dot plots showing the individual levels of SRC, CTRC and CCL17 in EV lysates. Mean, standard deviation and q-value are indicated. Receiver operating characteristic (ROC) curves comparing diagnostic accuracy of EV lysates (pink) and plasma (blue). Area under the curve (AUC) with 95% CI and p-value for testing the difference in AUC between plasma and EV is shown.

To identify proteins that were dysregulated in MI patients compared to controls, we performed multiple t-tests; followed by two-stage linear step-up to correct for multiple comparisons of all detected proteins. Volcano plots that show the differentially-expressed proteins (q < 0.01) in EV lysates and plasma is presented in Fig. 4a. In total, 11 and 47 proteins were differentially-expressed between patients and controls in EVs and plasma, respectively (Fig. 4c, Supplemental Table 3). 39 of the proteins were differentially-expressed in plasma only; 8 were common to both fractions and 3 were differentially-expressed in EVs only. Among the most dysregulated proteins in plasma were well-established biomarkers for cardiac injury including B–type natriuretic peptide (BNP) and interleukin-6 (IL-6). In addition, completely novel biomarker candidates such as agouti-related peptide (AGRP), chemokine (C motif) ligand (XCL1) and NF-kappa-B essential modulator (NEMO) were also evident. Proteins that were dysregulated in both plasma and EV lysates included growth factors (placental growth factor, PGF; platelet-derived growth factor, PDGFB), glycoproteins (angiopoietin 1, ANG-1) and enzymes (tissue transglutaminase, TGM2; glyoxalase I, GLO1). All have known roles in cardiovascular biology, but previous evidence as plasma biomarkers for MI is largely lacking. To ascertain whether the sample type affected the diagnostic accuracy of these identified biomarker candidates, ROC analysis was performed on these 8 proteins (Supplementary Fig. 3). Sensitivity and specificity was similar for the majority of the proteins, with area under the ROC curves (AUC) ranging from 0.68 to 0.99. The notable exceptions were IL-6 and PDGFB. IL-6 had a superior diagnostic accuracy in plasma (AUC-plasma: 0.99; AUC-EV: 0.90, p = 0.006) while PDGFB performed significantly better in EV lysates (AUC-plasma: 0.73; AUC-EV: 0.87, p = 0.01).

Lastly, the 3 proteins that were dysregulated in the EV fraction but not in plasma were chemokine (C-C motif) ligand 17 (CCL17), chymotrypsin C (CTRC) and proto-oncogene tyrosine-protein kinase SRC (SRC). Interestingly, all three were reduced in patients (between 1.2- and 2-fold, Fig. 4d), and neither CTRC nor SRC have been previously described as biomarkers for MI. Based on ROC analysis, CCL17 had significantly higher diagnostic accuracy in EV lysates compared to plasma (area under the curve (AUC) of 0.73 in EVs versus 0.51 in plasma, p = 0.01, Fig. 4d, Table 3). The AUCs of SRC and CTRC were numerically higher in EVs but were not statistically significant (Fig. 4d, Table 3). Using logistic regression and adjusting for age and sex, the association of each of the three EV-associated biomarkers with STEMI diagnosis was assessed. Independent of age and sex, each of the three proteins were associated with a 45–65% lower risk of STEMI diagnosis per standard deviation (Table 3).

**Table 3:** Diagnostic accuracy and association of EV-proteins with STEMI diagnosis.

Protein	Sample type	ROC-AUC	Sensitivity* (%)	Specificity* (%)	Fold change (CTRL vs STEMI)	FDR q	OR	95% CI	p
CTRC	EV	0.76	80	72.7	1.23	**0**.**003**	0.478	0.265–0.862	**0**.**014**
	Plasma	0.66	55	77.3	1.05	0.01	0.342	0.121–0.966	**0**.**043**
CCL17	EV	0.73	50	95.5	1.62	**0**.**004**	0.334	0.169–0.660	**0**.**002**
	Plasma	0.51	71.7	45.5	1.01	0.44	0.725	0.310–1.698	0.46
SRC	EV	0.74	83.3	59.1	1.96	**0**.**003**	0.540	0.328–0.888	**0**.**015**
	Plasma	0.64	48.3	81.8	1.06	0.05	0.56	0.244–1.303	0.18

Area under the Receiver Operating Characteristic curve (ROC-AUC) for each protein in EVs and plasma are presented. *Sensitivity and specificity at the optimal cut-off point as determined by Youden’s index. P-values adjusted for multiple comparisons (q) using a False discovery rate (FDR) approach comparing mean NPX values between STEMI patients and controls (CTRL) is presented. Results of binary logistic regression analyses are presented as odds ratios (OR) scaled to 1 standard deviation, 95% confidence intervals and p-values, adjusted for age and gender.

Taken together, these results suggest that although the majority of the detected, dysregulated proteins were observed was found in plasma, there is additional diagnostic and biological information in the proteome of isolated EVs.

### Validation of EV-associated proteins as biomarkers in patients with varying degrees of coronary artery disease

**Figure 5:**
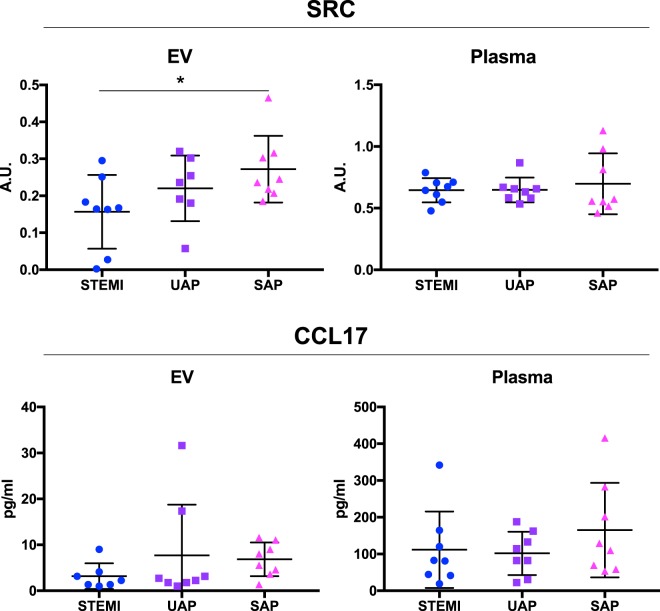
Validation of EV-associated proteins in patients with varying degrees of coronary artery disease. Plasma and acoustically trapped EVs were analyzed in patients diagnosed with ST-elevation myocardial infarction (STEMI), unstable angina pectoris (UAP) and stable angina pectoris (SAP) using SRC- and CCL17-ELISAs (n = 8 per group). One-way ANOVA with Dunnett’s multiple comparison test was used to test differences between patient groups. A.U. = arbitrary units. For the EV-SRC ELISA, data was missing from one patient in the UAP group. For the EV-CCL17 ELISA, one outlier in the STEMI group was detected and removed using the Robust regression and outlier removal method with a ROUT coefficient of Q = 0.01.

Next, we sought to validate these EV-associated proteins in a separate, clinically relevant patient material using the gold standard clinical diagnostic tool for the quantitation of protein biomarkers, the enzyme-linked immunosorbent assay (ELISA). We could identify commercially available, well-validated ELISAs for SRC and CCL17, but not CTRC, and used these to analyze plasma and acoustically trapped EV samples from patients with varying degrees of coronary artery disease. The validation cohort consisted of patients with stable angina pectoris (SAP), unstable angina pectoris (UAP) and STEMI (n = 8 per group, see Table 2 for patient characteristics). We observed a statistically significant decrease in EV-associated SRC in STEMI patients compared to SAP patients (Fig. 5, p < 0.05). A trend towards lower levels in STEMI patients compared to UAP was observed, but the effect was not statistically significant. Interestingly, the levels of SRC in plasma was unaltered across patient groups. For CCL17, no significant differences between groups were observed in either plasma or EVs.

These results confirm that EV-associated SRC, but not plasma SRC, is a marker of advanced coronary artery disease, but is not specific for MI.

### SRC is present on vesicles of different sizes and cellular origins

We perfomed a series of experiments in order to confirm that a fraction of circulating SRC is associated with EVs. First, we analyzed the levels of SRC in plasma from healthy donors (n = 5) before and after sequential depletion of microvesicles (obtained by centrifugation at 20,000 *g* for 1 h) and exosomes (obtained by centrifugation of microvesicle-depleted plasma at 100,000 *g* for 1 h) using ELISA (Figiure 6a). There was a 16% decrease in SRC levels after depletion of microvesicles (p < 0.05) and a further 7% decrease in the signal after depletion of exosomes (not statistically significant). These results suggest that approximately a quarter of circulating SRC is associated with EVs.

**Figure 6:**
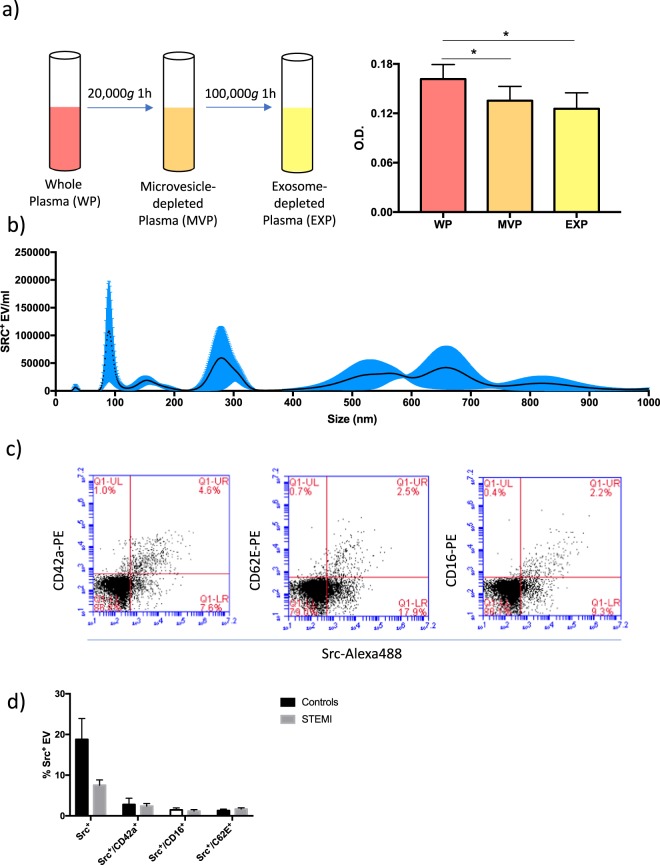
SRC is present on vesicles of different sizes and cellular origins. (**a**) Depletion of microvesicles and exosomes from normal plasma was obtained by sequential centrifugation at 20,000 × *g* for 1 h and 100,000 × *g* for 1 h. SRC was quantified in each plasma fraction using ELISA (n = 5). Repeated measures 1-way ANOVA was used to test the difference between whole plasma and microvesicle- and exosome-free plasma, respectively, *p < 0.05. (**b**) Nanoparticle tracking analysis was performed on trapped EVs isolated from a healthy individual and stained with a primary SRC antibody and a secondary Alexa488-conjugated antibody. The size distribution of fluorescently labeled EVs was measured. Mean and standard error from 5 repeated measurements are presented. (**c**) Acoustically trapped vesicles from one healthy control were co-stained with SRC/IgG-Alexa488 antibodies and PE-conjugated antibodies against either CD42a, CD62E or CD16. Gates were set to include particles in the size range 240–800 nm. Representative scatter plots showing the proportion of single- and double positive EVs for each staining are presented. (**d**) Proportion of SRC^+^ EV subpopulations in the plasma of healthy individuals and STEMI patients (n = 6 per group). Mean and standard deviation is indicated.

Next, the size distribution of acoustically trapped SRC^+^ EVs was investigated by fluorescent nanoparticle tracking analysis (NTA). Results revealed that SRC^+^ EVs were present across a broad range, with notable peaks at approximately 100, 280 and 600 nm (Fig. 6b). This provides further evidence that SRC is present on microvesicles as well as exosomes. The size distribution of SRC^+^ EVs isolated by centrifugation (20,000 *g* for 1 h) were largely similar, although an additional peak < 50 nm and a more prominent peak at 800 nm were observed (Supplementary Fig. 4a).

The main source of circulating microvesicles are platelets, endothelial cells and leukocytes. The cellular origin of acoustically trapped SRC^+^ microvesicles in blood samples from a healthy donor was investigated by co-staining for SRC and either of the cell surface markers CD42a (platelets), CD62E (endothelial cells) and CD16 (leukocytes). The proportion of single- and double positive events was then determined with flow cytometry. It is important to note that, due to the limited sensitivity of the flow cytometer, only vesicles > 200 nm was recorded in this experiment. As seen in Fig. 6c, there was a subset of SRC^+^ vesicles within each of the CD42a^+^, CD62E^+^ and CD16^+^ populations. This suggests that the source of SRC^+^ microvesicles in blood is not restricted to a particular cell type. When performing the same analysis on EVs isolated by centrifugation (20,000 *g* for 1 h), the proportion of SRC^+^ vesicles were substantially higher (40% of events in the EV gate compared to 20% for trapped samples) and SRC + /CD42a + EVs were slightly more prominent (Supplementary Fig. 4b), but the levels were not significantly higher than those of SRC + /CD62E + and SRC + /CD16 + vesicles (Supplementary Fig. 4c).

To investigate whether the decrease in EV-associated SRC was driven by a specific subpopulation of microvesicles, we analyzed the proportion of total SRC^+^ as well as SRC^+^/CD42a^+^, SRC^+^/CD62E^+^ and SRC^+^/CD16^+^ events in STEMI patients and healthy controls (n = 6 per group) using flow cytometry. Results showed that even though a general decrease in SRC^+^ EVs was observed in STEMI patients, neither of the SRC^+^ subpopulations were altered (Fig. 6d). These results indicate that the change in EV-associated SRC observed in STEMI patients is driven by additional, unidentified subpopulation(s) of EVs.

## Discussion

Mounting evidence suggests that the contents of circulating EVs mirror pathophysiological processes that have occurred in the cell or tissue of origin. Similarly, the EV proteome is increasingly considered a rich source of biomarkers for various disease states. To date, most studies that have explored the EV proteome have essentially applied a mass spectrometry-based approach. Under such circumstances the broad dynamic range of these proteomes leads to limited sensitivity and reproducibility^16^. Based on PEA technology, a highly-sensitive multiplex immunoassay was employed in this study to probe the EV proteome of patients with MI and healthy controls. Of the 92 proteins with relevance to cardiovascular disease included in the PEA analysis, 52 were detected in the EV lysates. Although the overall protein profile of isolated EVs largely reflected that of whole plasma, some striking differences were revealed. The levels of SRC, CCL17 and CTRC were all significantly decreased in EV lysates from MI patients but remained unaltered in the corresponding plasma samples.

CCL17 (C-C motif ligand 17) is produced by a wide range of cells (most notably dendritic cells, but also endothelial cells, keratinocytes and fibroblasts)^28^ and is known to regulate leukocyte migration and T-cell differentiation and expansion. Elevated expression of CCL17 has been reported both in the circulation^29^ and in atherosclerotic lesions of coronary artery disease patients^30^. Additionally, dendritic cell-derived CCL17 was shown to drive development of atherosclerosis in a mouse model^31^. In light of these reports, our finding that EV-associated CCL17 was decreased in STEMI patients was somewhat surprising. There are, however, reports that EVs facilitate clearance of certain proteins^32^, and it is possible that the decrease in EV-associated CCL17 can be explained by such a mechanism.

CTRC (chymotrypsin C) is a member of the peptidase S1 family of proteins. Activation and degradation of trypsinogens and procarboxypeptidases are regulated by CTRC, but very little is known about the role of this protein in cardiovascular biology, or the potential association with EVs.

SRC is a non-receptor tyrosine kinase that regulates a wide array of signaling pathways^33^. This kinase has been shown to be an important factor in cardiovascular disease mechanisms, primarily via the key role played in vascular physiology^34^, platelet activation^35^ and inflammation^36^. One previous study had described the presence of SRC on vesicles derived from endothelial and cardiomyocyte cell lines and also in plasma vesicles from patients with amyloidosis^37^, but the presence and potential role of EV-associated SRC in cardiovascular disease has not been studied before. Our results show that decreased levels of vesicle-associated SRC is not specific to myocardial infarction, but seems rather to be a reflection of advanced coronary artery disease. Interestingly, although circulating SRC was found to be present on vesicles of different cellular origins, we were unable to identify a specific subpopulation of EVs that drives the decrease in total SRC^+^ EVs in MI patients. We believe a more comprehensive investigation of the potential mechanistic role for SRC in the progression of coronary artery disease is warranted.

The use of EVs as a source of biomarkers for clinical use is hampered by the lack of large-scale, high-throughput isolation techniques. EVs are typically isolated from plasma using high-speed or ultracentrifugation, which is time-consuming, requires large sample volumes and can produce artefactual EV-like particles^38^. The acoustic trapping platform used in this study has several advantages which in our view makes it a better alternative for clinical use. First, EV isolation can be performed within minutes (the protocol used here was finished in approximately 7 minutes, including washing), while a typical centrifugation protocol takes 1 hour (2 hours if washing is necessary). Second, while centrifugation requires manual pipetting steps, trapping is fully automated, which should reduce variability and increase clinical applicability. The ability to automate the trapping process also facilitates continuous, large-scale sample handling, a requirement for use in a clinical context. Third, the miniaturized flow-through format also enables integration with other miniaturized techniques, e.g. integrating a plasmapheresis system upstream^39^ in order to isolate EVs directly from whole blood and further reduce sample processing time.

One concern regarding the analysis of EV cargo (proteins, miRNA, *etc*.) as biomarkers is that the results will be different depending on isolation technique. When it comes to EV proteomics, we have shown in previous reports that there is considerable overlap in the protein profiles of EVs that were isolated with acoustic trapping and standard centrifugation^15,27^. Importantly, we show here that the PEA-based proteomic profiles of EVs isolated with acoustic trapping is also very similar to that of EVs isolated with centrifugation.

Another concern regarding EV isolation is that different techniques and protocols will capture or enrich specific EV subpopulations, differing in size and/or content. Recently, two different groups used nanoparticle tracking analysis to show that acoustic trapping of plasma captures EVs of a wide size range, similar to standard centrifugation protocols^27,40^. Comparing the outcomes of our flow cytometric analyses on EVs isolated by trapping and centrifugation, there were some distinct differences. The proportion of total SRC^+^ and SRC^+^/CD42a^+^ vesicles were higher in the centrifuged samples, indicating that these EV subpopulations might be more efficiently isolated using centrifugation. However, a more thorough study would be needed in order to prove or reject this hypothesis.

Despite a clear outcome, our study does have some limitations. First, the 92 proteins that were assayed are likely to only represent a fraction of the EV proteome, and the assay panel used was specifically focused on proteins with known roles in cardiovascular biology/pathobiology. An unbiased approach, *e*.*g*. an aptamer-based technique^41^, would yield a considerably more comprehensive profile of the EV proteome during MI. Second, the sample size of this study is rather limited, and validation was performed in a small patient material. As such the findings of this study are more exploratory in nature, and the diagnostic and prognostic value of these EV-associated markers will have to be confirmed in larger validation studies. Nevertheless, based on the results of our work, analysis of EV-associated proteins in larger patient cohorts is well-warranted.

## Supplementary information

Supplementary Information

## Data Availability

The datasets generated during or analyzed during the current study are available from the corresponding author on reasonable request.
