# Supplementary material for: Proteomic profiling of extracellular vesicles reveals additional diagnostic biomarkers for myocardial infarction compared to plasma alone
Source: Sci Rep. 2019 Jun 20;9:8991. doi: 10.1038/s41598-019-45473-9 (PMC6586849; doi:10.1038/s41598-019-45473-9)
Supplement: Supplementary file 1 — Supplementary Information [file 41598_2019_45473_MOESM1_ESM.pdf]

Supplementary Information

Proteomic profiling of extracellular vesicles reveals additional diagnostic biomarkers for myocardial infarction compared to plasma alone

Olof Gidlöf, Mikael Evander, Melinda Rezeli, György Marko-Varga, Thomas Laurell, David Erlinge

Supplementary Figure 1-4
Supplementary Table 1-3

Supplementary Figure 1

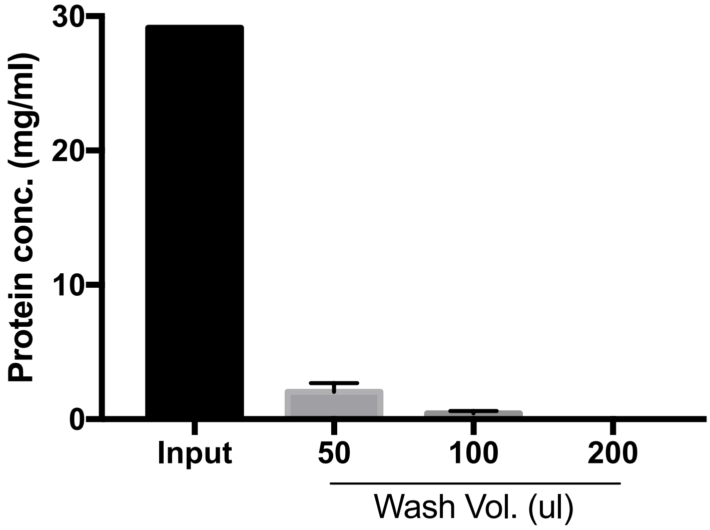

Supplementary Figure 1. Plasma was diluted 1:1 with PBS and used for acoustic seed trapping with increasing washing volumes (n=3). The amount of soluble protein in input plasma and each trapped fraction was measured using Nanodrop at 280 nm.

Supplementary Figure 2

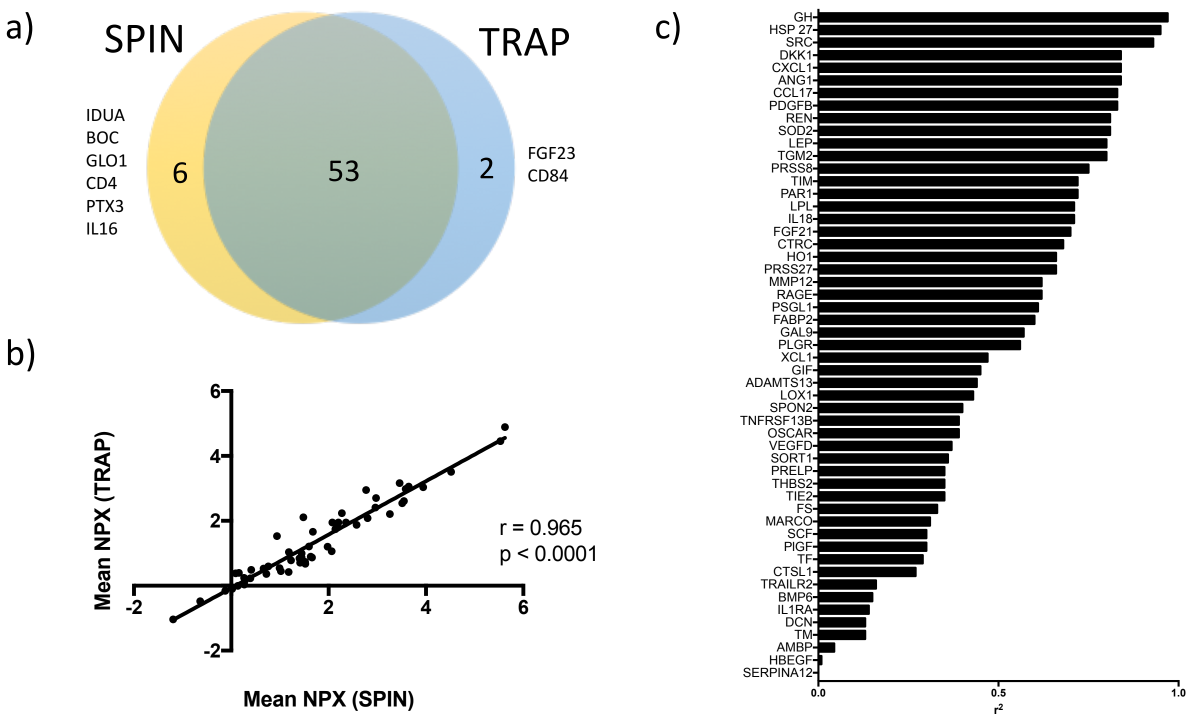

Supplementary Figure 2. Blood samples were drawn from 10 healthy individuals before, during and after a standardized exercise program (Bryl-Gorecka et al 2018). EVs were isolated from plasma with acoustic trapping or centrifugation (20,000x*g* 1h) and analyzed with the Olink CVD II PEA Panel. A) Detected proteins (i.e. proteins with a signal above the limit of detection in >50% of samples) in EVs isolated with centrifugation (SPIN, yellow, n=59) and acoustic trapping (TRAP, blue, n=55). Non-overlapping proteins for each fraction are named. B) Correlation of the mean protein signal between trapped and centrifuged EVs. Correlation coefficient and p-value are presented. C) Correlation coefficients for each individual protein comparing trapped and centrifuged samples. Statistically significant correlations (p<0.05) were observed for all proteins except AMBP, HBEGF and SERPINA12.

Supplementary Figure 3

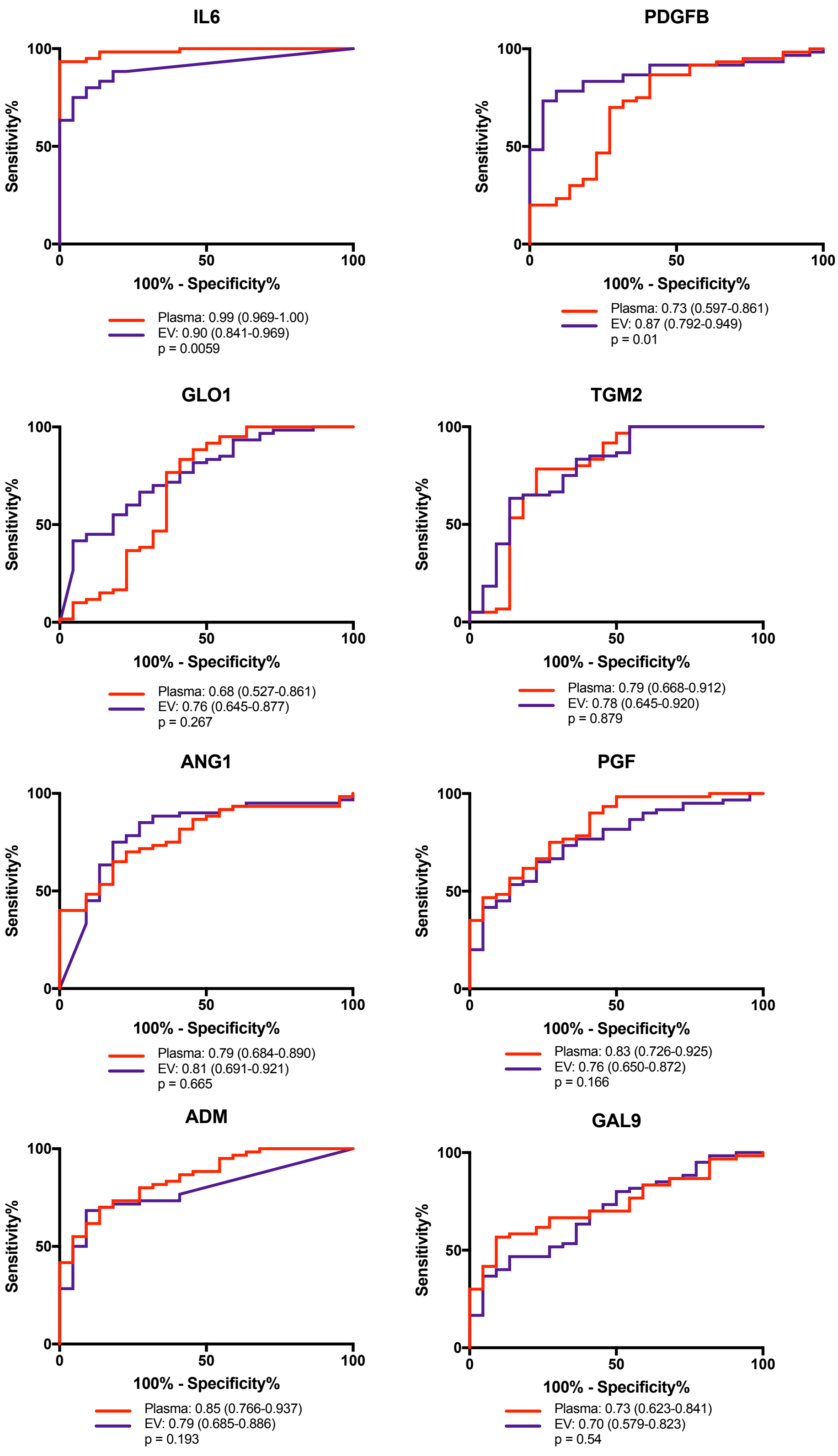

Supplementary Figure 3. Comparison of diagnostic accuracy across sample types. ROC curves of the 8 proteins that were differentially-expressed in EVs (purple) and plasma (red). Area under the curve (AUC) with 95% CI and p-value for testing the difference in AUC between plasma and EV is shown.

Supplementary Figure 4

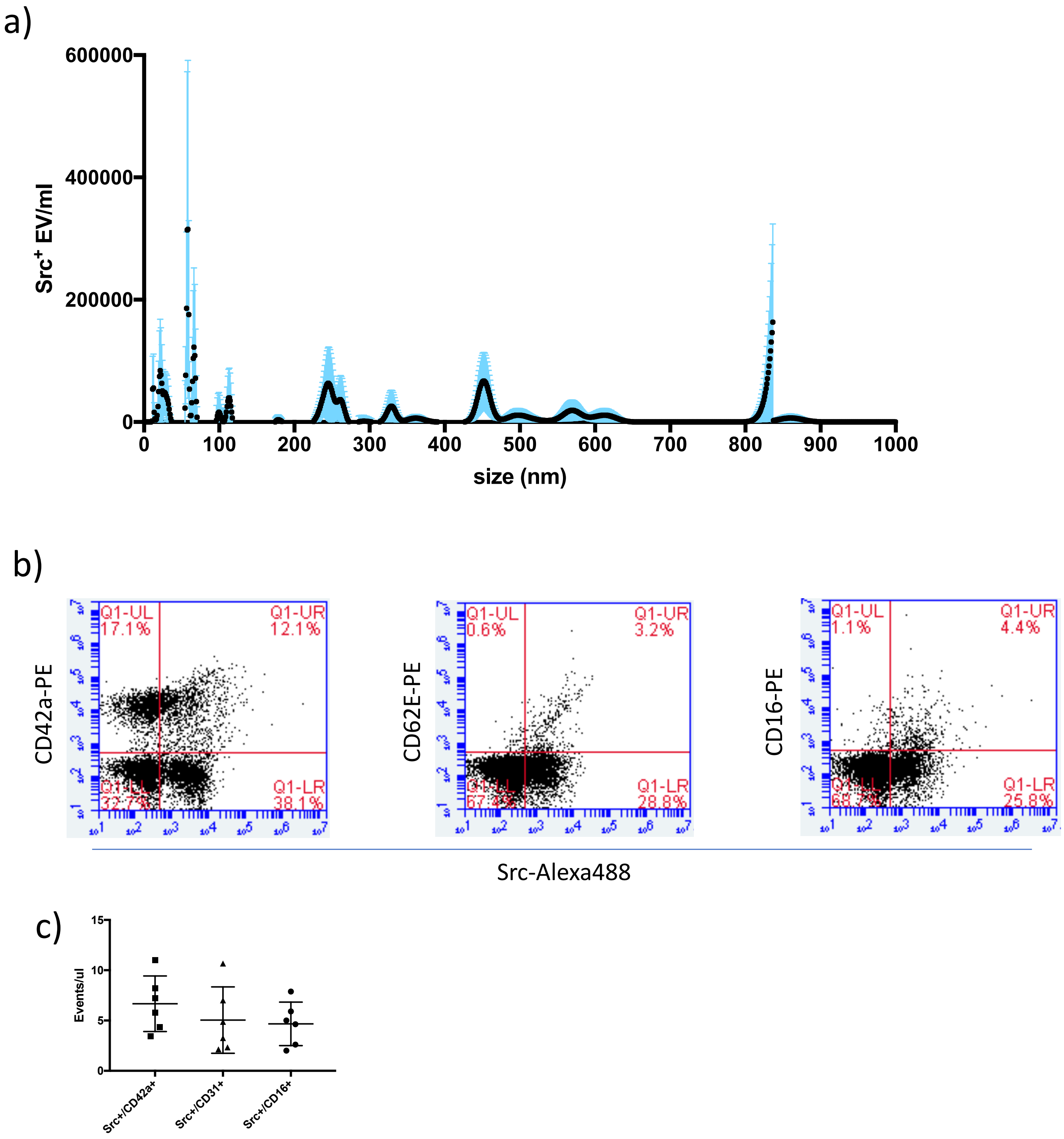

Supplementary Figure 4. A) Nanoparticle tracking analysis was performed on EVs isolated from a healthy individual using centrifugation (20,000*g* for 1 h), stained with a primary SRC antibody and a secondary Alexa488-conjugated antibody. The size distribution of fluorescently labeled EVs was measured. Mean and standard error from 5 repeated measurements are presented. (B) EVs isolated using centrifugation (20,000*g* for 1 h), from healthy controls (n=6) were co-stained with SRC/IgG-Alexa488 antibodies and PE-conjugated antibodies against either CD42a, CD62E or CD16. Representative scatter plots from one individual showing the proportion of single- and double positive EVs for each staining. C) Concentration of double- positive EVs in the plasma of healthy individuals. Mean and standard deviation is indicated.

Supplemental Table 1 - Proteins on PEA Panel

Gene symbol	Protein name
DECR1	2,4-dienoyl-CoA reductase, mitochondrial
ADAMTS13	A disentigrin and metalloproteinase with a thrombospondin type 1 motif, member 13
ADM	Adrenomedullin
AGRP	Agouti-related protein
IDUA	Alpha-L-iduronidase
ANG-1	Angiopoietin 1
TIE2	Angiopoietin-1 receptor
ACE2	Angiotensin-converting enzyme 2
BMP6	Bone morphogenetic protein 6
BOC	Brother of CDO
CCL17	C-C motif chemokine 17
CCL3	C-C motif chemokin 3
CXCL1	C-X-C motif chemokine 1
CA5A	Carbonic anhydrase 5A, mitochondrial
CEACAM8	Carcinoembryonic antigenrelated cell adhesion molecule 8
CTSL1	Cathepsin L1
CD40L	CD40 Ligand
CTRC	Chymotrypsin C
DCN	Decorin
DKK1	Dickkopf-related protein 1
FABP2	Fatty acid-binding protein, intestinal
FGF21	Fibroblast growth factor 21
FGF23	Fibroblast growth factor 23
FS	Follistatin
GAL9	Galectin-9
GIF	Gastric intrinsic factor
GT	Gastrotropin
GH	Growth hormone
GDF2	Growth/differentiation factor 2
HSP27	Heat shock 27 kDa Protein
HO1	Heme oxygenase 1
HAOX1	Hydrocyacid oxidase 1
IL1RA	Interleukin-1 receptor antagonist protein
IL1RL2	Interleukin-1 receptor-like 2
IL17D	Interleukin-17D
IL18	Interleukin-18
IL27	Interleukin-27
IL6	Interleukin-6
KIM1	Kidney Injury Molecule
GLO1	Lactoylglutathione lyase
LOX1	Lectin-like oxidized LDL receptor 1
LEP	Leptin
LPL	Lipoprotein lipase

IgG Fc receptor II-b	Low affinity immunglobulin gamma Fc region receptor II-b
XCL1	Lymphotactin
MARCO	Macrophage receptor MARCO
MMP12	Matrix metalloproteinase-12
MMP7	Matrix metalloproteinase-7
ITGB1BP2	Melusin
BNP	B-type natriuretic peptide
NEMO	NF-kappa-B essential modulator
OSCAR	Osteoclast-associated immunoglobulin-like receptor
PSGL1	P-selectin glycoprotein ligand 1
PAPPA	Pappalysin-1
PTX3	Pentraxin-related protein 3
PGF	Placental growth factor
PDGFB	Platelet-derived growth factor subunit B
PARP1	Poly(ADP-ribose) polymerase-1
PLGR	Polymeric immunoglobulin receptor
IL16	Interleukin-16
PDL2	Programmed cell death 1 ligand 2
HBEGF	Proheparin-binding EGF-like growth factor
PRELP	Prolargin
PRSS8	Prostatin
AMBP	Alpha-1-microglobulin/bikunin precursor
TGM2	Transglutaminase 2
PAR1	Proteinase-activated receptor 1
SRC	Proto-oncogene tyrosine-protein kinase Src
RAGE	Receptor for advanced glycosylation end products
REN	Renin
PRSS27	Serine protease 27
STK4	Serine/threonin-protein kinase 4
SERPINA12	Serpin A12
CD84	SLAM family member 5
SLAMF7	SLAM family member 7
SORT1	Sortilin
SPON2	Spondin-2
SCF	Stem cell factor
SOD2	Superoxide dismutase, mitochondrial
CD4	T-cell surface glycoprotein CD4
TM	Thrombomodulin
THPO	Thrombopoietin
THBS2	Thrombospondin-2
TF	Tissue factor
TRAILR2	TNF-related apoptosis-inducing ligand receptor 2
TNFRSF10A	Death receptor 4
TNFRSF11A	Receptor activator of nuclear factor kappa B
TNFRSF13B	Transmembrane activator and CAML interactor

MERTK	Tyrosine-protein kinase Mer
VSIG2	V-set immunoglobulin domain-containing protein 2
VEGFD	Vascular endothelial growth factor D

Supplemental Table 2 - Overlapping samples from PCA

EV		Plasma	
**Overlapping**	**Non-overlapping**	**Overlapping**	**Non-overlapping**
C1	C14	C1	C3
C2	C15	C2	C4
C3	C16	C4	C6
C4	C17	C5	C17
C5	C18	C7	C18
C6	C19	C8	C20
C7	C20	C9	C22
C8	C21	C10	
C9	C22	C11	S1
C10		C12	S2
C11	S10	C13	S3
C12	S15	C14	S5
C13	S29	C15	S6
	S3	C16	S7
S1	S46	C19	S8
S2	S5	C21	S9
S4	S50		S10
S5	S60	S4	S12
S6	S8	S11	S13
S7		S15	S14
S9		S17	S16
S10		S19	S18
S12		S22	S20
S13		S23	S21
S14		S24	S25
S15		S29	S26
S16		S30	S27
S17		S31	S28
S18		S35	S32
S19		S37	S33
S20		S39	S34
S21		S40	S36
S22		S47	S38
S23		S50	S41
S24		S51	S42
S25		S52	S43
S26		S53	S44
S27		S54	S45
S28		S55	S46
S30		S58	S48
S31		S59	S49
S32			S56

S33			S57
S34			S60
S35			
S36			
S37			
S38			
S39			
S40			
S41			
S42			
S43			
S44			
S45			
S47			
S48			
S49			
S51			
S52			
S53			
S54			
S55			
S56			
S57			
S58			
S59			

C=Control, S=STEMI-patient

Supplemental Table 3 - All Discoveries

**EV**					
**Gene symbol**	**P value**	**Mean STEMI**	**Mean CTRL**	**Difference**	**q value**
PDGFB	3,12001E-08	2,151	3,752	-1,601	1,32351E-06
GLO1	2,5056E-05	0,611	1,064	-0,4528	0,000531438
TGM2	4,66666E-05	2,782	2,031	0,7507	0,000566885
ANG-1	5,34545E-05	1,089	1,941	-0,8514	0,000566885
IL6	0,000180633	1,91	1,393	0,5168	0,001532486
PGF	0,000245783	1,901	1,6	0,3009	0,001737684
ADM	0,000319567	1,181	0,9482	0,2323	0,001936574
SRC	0,000573606	0,5312	1,04	-0,5093	0,003015953
CTRC	0,000639877	2,13	2,634	-0,5043	0,003015953
CCL17	0,001017116	1,513	2,441	-0,9279	0,004314606
GAL9	0,002314628	4,066	3,631	0,4356	0,008926047
**Plasma**					
**Gene Symbol**	**P value**	**Mean STEMI**	**Mean CTRL**	**Difference**	**q value**
IL6	1,00E-14	6,432	3,648	2,784	2,1E-14
PTX3	2,48E-13	2,98	1,649	1,332	5,882E-12
AGRP	1,2315E-11	4,059	2,879	1,18	1,94858E-10
BNP	8,3837E-11	4,437	1,37	3,067	9,9494E-10
ADAM-TS13	1,23985E-10	5,136	5,582	-0,4462	1,17712E-09
CTSL1	3,18372E-09	7,32	6,492	0,8279	2,3189E-08
IL-1ra	3,41948E-09	2,362	1,265	1,097	2,3189E-08
PARP-1	5,93063E-09	2,809	1,836	0,9733	3,51909E-08
OSCAR	1,1464E-08	10,35	10,03	0,3235	6,04662E-08
CD4	2,51804E-08	5,121	4,441	0,6792	1,149E-07
ADM	2,66253E-08	8,294	7,125	1,169	1,149E-07
TRAIL-R2	3,32574E-08	5,776	4,909	0,8667	1,31561E-07
TGM2	3,79279E-08	7,772	6,335	1,436	1,38495E-07
NEMO	6,07406E-08	3,99	5,168	-1,179	2,05954E-07
LOX-1	2,7825E-07	6,738	5,938	0,7996	8,80569E-07
PGF	9,72315E-07	8,214	7,678	0,5359	2,88474E-06
CEACAM8	1,05932E-06	4,13	3,196	0,9336	2,95799E-06
XCL1	1,72032E-06	5,395	4,611	0,784	4,53688E-06
TNFRSF10A	6,31882E-06	3,372	2,785	0,5872	1,57871E-05
IL-27	1,03124E-05	5,004	4,408	0,5954	2,44765E-05
SOD2	1,60385E-05	8,94	8,689	0,2508	3,62547E-05
RAGE	3,22937E-05	5,015	4,536	0,4784	6,96809E-05
GLO1	5,95286E-05	5,718	6,728	-1,01	0,000122862
CCL3	7,79416E-05	6,443	5,338	1,105	0,000154162
KIM1	0,000154164	8,611	7,733	0,8784	0,000292726
ANG-1	0,000208602	6,511	7,57	-1,059	0,000380858
TNFRSF11A	0,00021793	5,988	5,347	0,6413	0,000383153

TNFRSF13B	0,000232135	8,87	8,195	0,6755	0,000390497
GT	0,00023856	1,66	1,05	0,6101	0,000390497
AMBP	0,000269679	7,523	7,33	0,1924	0,000426723
PDGFB	0,00032597	8,883	9,845	-0,9623	0,000499155
THPO	0,000337199	2,513	2,225	0,2881	0,000500214
FS	0,000814373	11,45	10,96	0,4975	0,001171463
MERTK	0,00120261	6,262	5,85	0,4126	0,001675442
SORT1	0,00126331	8,089	7,854	0,2358	0,001675442
ACE2	0,001301264	4,386	3,819	0,5663	0,001675442
SPON2	0,001317948	10,19	10,1	0,09814	0,001675442
PRELP	0,001341201	6,409	6,179	0,2295	0,001675442
MMP12	0,001763485	7,304	6,519	0,7858	0,002146478
LEP	0,001809144	6,382	5,529	0,8531	0,002147002
SLAMF7	0,001863508	3,722	3,154	0,5682	0,002157579
GDF-2	0,0019465	6,961	7,341	-0,3797	0,002200008
GAL9	0,002002475	8,673	8,368	0,3049	0,002210639
PAR-1	0,002886351	8,32	8,043	0,2763	0,003113979
TM	0,004386778	9,631	9,385	0,2467	0,004627564
FGF-23	0,007535767	3,138	2,626	0,5116	0,007776584
IL16	0,008200741	5,968	5,611	0,3572	0,008282749
